# Progress in the Understanding of the Mechanism of Tamoxifen Resistance in Breast Cancer

**DOI:** 10.3389/fphar.2020.592912

**Published:** 2020-12-09

**Authors:** Jingwei Yao, Kun Deng, Jialu Huang, Ruimin Zeng, Jianhong Zuo

**Affiliations:** ^1^ Nanhua Hospital Affiliated to University of South China, Hengyang, China; ^2^ Transformation Research Lab, Hengyang Medical School, University of South China, Hengyang, China; ^3^ The Third Affiliated Hospital of University of South China, Hengyang, China

**Keywords:** tamoxifen, breast cancer, cell cycle regulators, autophagy, resistance

## Abstract

Tamoxifen is a drug commonly used in the treatment of breast cancer, especially for postmenopausal patients. However, its efficacy is limited by the development of drug resistance. Downregulation of estrogen receptor alpha (ERα) is an important mechanism of tamoxifen resistance. In recent years, with progress in research into the protective autophagy of drug-resistant cells and cell cycle regulators, major breakthroughs have been made in research on tamoxifen resistance. For a better understanding of the mechanism of tamoxifen resistance, protective autophagy, cell cycle regulators, and some transcription factors and enzymes regulating the expression of the estrogen receptor are summarized in this review. In addition, recent progress in reducing resistance to tamoxifen is reviewed. Finally, we discuss the possible research directions into tamoxifen resistance in the future to provide assistance for the clinical treatment of breast cancer.

## Introduction

Breast cancer is the most common cancer in women (Bray et al., 2018), and endocrine therapy plays an important role in breast cancer treatment (Rugo et al., 2016). More than 60% of breast cancers are estrogen-receptor (ER) positive (Lopez-Tarruella and Schiff, 2007; Vargo-Gogola and Rosen, 2007). Tamoxifen is an antagonist of ERα66, and it is commonly used in the treatment of ER-positive breast cancers (Binkhorst et al., 2012); however, the efficacy is not satisfactory because of the development of tamoxifen resistance. RTKs (receptor tyrosine kinases) and the activation of the PI3K-PTEN/AKT/mTOR pathway caused by the overexpression of RTKs are thought to be closely related to resistance to tamoxifen (Hosford and Miller, 2014; Yin et al., 2014).

On the other hand, ERα36, a 36 kDa truncated isoform of ERα66 located on the cytoplasmic membrane of breast cancer (Lv et al., 2015; Omarjee et al., 2017), has been reported to be related to the drug resistance and metastasis of cancer cells (Zhang and Wang, 2013; Yin et al., 2014; Omarjee et al., 2017). Tamoxifen can activate ERα36, which in turn activates MAPK, AKT, and other signaling pathways, leading to tamoxifen resistance (Tong et al., 2010).

In recent years, a large body of evidence has shown that protective autophagy, cell cycle regulators, and some transcription factors play a key role in tamoxifen resistance, such as KLF4 regulating drug resistance by regulating MAPK and the discovery of LEM4 (Gao et al., 2018; Jia et al., 2018). Scientists have proposed many methods to reduce drug resistance through these mechanisms and have made great progress.

In this review, the development of tamoxifen resistance in breast cancer is discussed, with special emphasis on the effects of some newly discovered enzymes and transcription factors on tamoxifen resistance, the protective autophagy of cells, and the latest progress in cell cycle regulators.

### The Role of Receptor Tyrosine Kinases (RTKs) in Tamoxifen Resistance

RPTKs are a class of enzyme-linked receptors that have been found to come in many kinds, including epidermal growth factor (EGF) receptor, platelet-derived growth factor (PDGF) receptor, macrophage colony stimulating factor (M-CSF), insulin and insulin-like growth factor-1 (IGF-1) receptor, vascular endothelial growth factor (VEGF) receptor, and hepatocyte growth factor (HGF) receptor. The PI3K/AKT/mTOR signaling pathway is one of the important mechanisms of tamoxifen resistance, and HER2 activates PI3K as a member of the EGFR family (Mansouri et al., 2018a). It has been proven that high expression of *p*-AKT is associated with a worse prognosis, and inhibiting the expression of AKT is beneficial for sensitizing drug-resistant cells (Block et al., 2012; Karlsson et al., 2019). In addition, activation of the PI3K/AKT pathway is not just associated with tamoxifen resistance. Recent studies have shown that activation of the PI3K/AKT pathway can cause tamoxifen-resistant (TAM-R) cells to develop drug resistance to DNA-damaging chemotherapy by upregulating BARD1 and BRCA1 (Zhu et al., 2018), which makes the PI3K/AKT pathway particularly important in the treatment of breast cancer.

The mechanism of activation of the PI3K/AKT/mTOR pathway has also been studied by many scientists. CC chemokine ligand 2 (CCL2), which is secreted by tumor-associated macrophages (TAMs), has been found to be related to activation of the PI3K/AKT/mTOR pathway. However, NF-κB promotes the secretion of CCL2 (Li et al., 2020a). Inhibition of the PI3K/AKT pathway may be beneficial to improve the efficacy of chemotherapy and endocrine therapy for breast cancer patients. Many drugs targeting PI3K, mTOR, or AKT to overcome tamoxifen resistance have been put into use. However, due to the complexity of the PI3K/AKT/mTOR pathway, inhibiting the pathway at any level will activate compensatory mechanisms, which limits the efficacy of inhibitors (Choi et al., 2016; Lui et al., 2016). We need to study the cross-talk between these pathways in future research.

The combined use of several inhibitors may be an important way to improve tamoxifen resistance in the future. Both VEGF and HER2 are members of the RTK family. Studies have shown that the expression of VEGF in drug-resistant cells is upregulated. VEGF contributes to angiogenesis and promotes tumor growth, which is not conducive to a good prognosis of breast cancer patients (Oh et al., 2010). The MAPK/ERK pathway has been proven to contribute to tamoxifen resistance (Heckler et al., 2014; Peng et al., 2017; Yin et al., 2017), whereas VEGF overexpression in drug-resistant cells leads to increased activation of MAPK. Surprisingly, the use of VEGF inhibitors was not found to be helpful in overcoming tamoxifen resistance (Mansouri et al., 2018b), which may also be attributed to the complex network of drug resistance. There is still no evidence that VEGF is related to tamoxifen resistance.

EGFR is also thought to be related to tamoxifen resistance. Tamoxifen downregulates the expression of miR-186-3p, which leads to further upregulation of the expression of EREG, a target gene of miR-186-3p. EREG then activates EGFR even more, subsequently enhancing glycolysis and leading to tamoxifen resistance (He et al., 2019). It has been reported that the NOGO-B receptor is related to tamoxifen resistance. The NOGO-B receptor contributes to the transport of RAS, which enhances EGF signal transduction, resulting in a decrease in p53 expression and the development of drug resistance (Gao et al., 2018).

ERα36 has been reported to be associated with tamoxifen resistance (Yin et al., 2014), and ERα36 reduces the sensitivity of breast cancer cells to tamoxifen by upregulating EGFR. EGFR expression and the basal level of ERK phosphorylation are upregulated in TAM-R cells. The EGFR/ERK signaling pathway can be blocked by knocking out ERα36 (Li et al., 2020b). However, lapatinib cannot only inhibit the phosphorylation of EFGR and HER2, but also decreases the expression of ERα36 (Yin et al., 2014). Interestingly, studies have shown that cross-talk between HER2 and ERK is conducive to the development of drug resistance (Ito et al., 2012).

In addition to members of the RTK family, such as HER2, EGFR, and VEGF, some research has shown that IGFR is also associated with tamoxifen resistance. Inhibition of IGF-1R reduces the sensitivity of cells to tamoxifen, which may be due to the inhibition of FoxO1 expression by the reduction of IGF-1R expression (Vaziri-Gohar et al., 2017). However, IGF1R signaling may be beneficial to the development of tamoxifen resistance in some aspects. P21-activated kinase 2 (PAK2) is a tamoxifen resistance inducer, while IGF1R can lead to the development of tamoxifen resistance by promoting the expression of PAK2 (Zhang et al., 2018).

**FIGURE 1:**
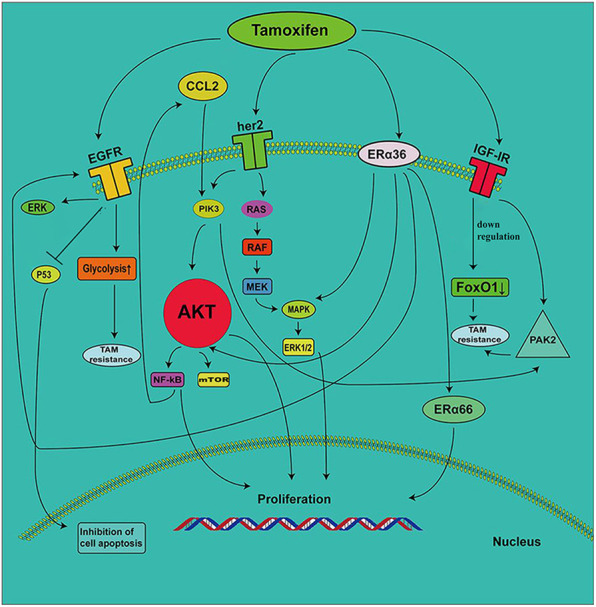
The role of RTKs and ERα36 in the development of tamoxifen resistance. EGFR induces tamoxifen resistance by enhancing the glycolytic pathway. The increase in EGF signal transduction induces a decrease in P53 expression, which leads to the inhibition of cell proliferation. TAMs secrete CC-chemokine ligand 2 (CCL2), which activates the PI3K/AKT/mTOR pathway. NF-κB promotes the secretion of CCL2. ERα36 contributes to the upregulation of EGFR, which increases ERK phosphorylation. The decrease in IGF-1R expression leads to the inhibition of FoxO1 expression, which results in the development of tamoxifen resistance. IGF1R mediates the expression of PAK2 and leads to drug resistance.

In general, there is a complex network in the mechanisms of action of the RTK family, and ERα36 affects the sensitivity of breast cancer cells to tamoxifen. These signaling cascades are described in Figure 1. The development of inhibitors for corresponding targets based on these mechanisms is the focus of previous research. However, due to the compensatory mechanisms that appear when any specific target is inhibited, the clinical effect of improving drug resistance is not very significant. Therefore, studies on improving drug resistance by other mechanisms have emerged in recent years.

### The Role of Enzymes and Transcription Factors in Tamoxifen Resistance

Based on the aforementioned mechanism, some enzymes and transcription factors also play a vital role in the complex network of ER-positive breast cancer resistance to tamoxifen. SOX9 is a transcription factor related to endocrine resistance (Jeselsohn et al., 2017; Xue et al., 2019). Histone deacetylase 5 (HDAC5), a member of the HDAC family whose main function is to remove acetyl groups, enables the deacetylation of SOX9 to facilitate its nuclear localization in TAM-R cells. Moreover, MYC plays an important role in the activation of HDAC5 transcription, and the C-MYC/HDAC5/SOX9 axis is related to tamoxifen resistance (Xue et al., 2019).

HDAC1, another member of the HDAC family, has also been reported to be associated with tamoxifen resistance. The expression of RBP2 is significantly higher in TAM-R cells than in cells sensitive to tamoxifen. The RBP2–ER–NRIP1–HDAC1 complex leads to IGF1R activation. The relationship between RBP and tamoxifen resistance is related to the PI3K/AKT pathway. RBP activates the PI3K/AKT pathway by enhancing the cross-talk between IGF1R and the HER2 receptor, which leads to drug resistance (Choi et al., 2018). Interestingly, it has also been reported that HDAC promotes the expression of ERα66 and AKT, and the use of HDAC inhibitors can inhibit the level of AKT by reducing the stability of its mRNA (Thomas et al., 2013).

Silent information regulator 2-related enzyme 1 (SIRT1) is a deacetylase dependent on nicotinamide adenine dinucleotide, which is highly expressed in a variety of tumors and has been proven to inhibit the growth of breast cancer cells (Liu et al., 2009; Kuo et al., 2013). The T-box protein Brachyury, a transcription factor, promotes the resistance of breast cancer cells to tamoxifen by inhibiting SIRT1 (Li et al., 2016). There are many different mechanisms for the effects of acetylases on tamoxifen resistance.

Estrogen regulates tumor growth by binding to ERα66 in the cytoplasm. Tamoxifen is antagonistic to ERα66. However, the use of tamoxifen has been confirmed to be involved in the upregulation of ERα36, SPhk1 (sphingosine kinase 1), and S1P (sphingosine-1-phosphate), which further activates downstream signaling pathways and causes drug resistance (Maczis et al., 2018), while the inhibition of ERα36 is beneficial to restore the sensitivity of breast cancer cells to tamoxifen.

Protein arginine N-methyltransferase 2 (PRMT2; HRMT1L1) is a member of the arginine methyltransferase family (Scott et al., 1998) that inhibits the resistance of breast cancer cells to tamoxifen by inhibiting the ERα36, PI3K, MAPK, and other signaling pathways (Shen et al., 2018).

It has been shown that the expression of hypoxia inducible factor HIF-1α contributes to the decrease in ERα, which is related to the sensitivity of endocrine therapy. HIF-1α reduces the sensitivity of breast cancer cells to tamoxifen. Interestingly, the expression of HIF-1α is related to the expression of EGFR (Jogi et al., 2019).

In contrast to HIF-1α, Spalt-like transcription factor 2 (SALL2), a transcription factor related to disease progression, enhances the sensitivity of breast cancer cells to tamoxifen, while ERα is downregulated after silencing SALL2 (Ye et al., 2019). This shows that tamoxifen is an effective endocrine therapy drug in ER-positive breast cancer patients. However, the expression of ERα is positively correlated with the sensitivity of tamoxifen therapy in ER-positive breast cancer patients. Numerous transcription factors regulate the sensitivity of breast cancer cells to tamoxifen by regulating ERα through various mechanisms. In addition, ERα also mediates the expression of glutathione S-transferase mu 3 (GSTM3) to resist cytotoxicity caused by drug therapy and to protect the drug-resistant cells.

The expression of GSTM3 was found to be higher in HER2-positive cancer cells (Lin et al., 2018). This indicates that there may be a relationship between GSTM3 and the RTK pathway, and the mechanism by which enzymes and transcription factors regulate tamoxifen resistance is also closely related to the RTK pathway. Li et al. (2018) found that the ER–c-Src–HER2 complex plays a vital role in tamoxifen resistance, while c-Cbl reverses tamoxifen resistance by inhibiting the formation of the ER–c-Src–HER2 complex. It seems that most enzymes are involved in drug resistance through the RTK pathway.

In addition, some enzymes can be used to predict the sensitivity of endocrine therapy in breast cancer. Shimoda et al. (2017) found that the expression of ASPH was upregulated in tamoxifen-resistant cells, and the upregulation depended on the PI3K and MAPK pathways. The cells with high expression of ASPH were more sensitive to tamoxifen than those with low expression of ASPH, and the results were statistically significant.

Aspartate-b-hydroxylase (ASPH) may also predict the sensitivity of breast cancer cells to tamoxifen (Shimoda et al., 2017). Gwak et al. (2017) also found that the expression of the transcription factor OCT 4 may be related to the poor efficacy of tamoxifen, and its expression level can be used to predict the sensitivity of breast cancer cells to tamoxifen. The mechanisms mentioned in this review related to tamoxifen resistance are summarized in Table 1.

**TABLE 1:** Summary of mechanisms leading to tamoxifen resistance

Factors	Mechanism(pathway)	Expression in breast cancer	Ref
EREG	miR-186-3p/EREG/EGFR regulatory cascade	High	(He et al., 2019)
NgBR	Promote EGF signaling	High	(Gao et al., 2018)
ERα36	Promote EGFR/ERK signaling	High	(Li et al., 2020b)
	Activate HER2 expression and its cascade	High	(Mansouri et al., 2018b)
	Activate Sphkl/S1P axis	High	(Maczis et al., 2018)
IGF1R	Inhibit FoxOl expression	Low	(Vaziri-Gohar et al., 2017)
PAK2	PAK2 acts downstream of IGF1R signaling	High	(Zhang et al., 2018)
Brachyury	Downregulate SIRT1 expression	High	(Li et al., 2016)
RBP2	Activate ER-IGF1R-ErbB signaling cascade	High	(Choi et al., 2018)
Cyclin D1	Promote the progress of G1-S phase	High	(Viedma-Rodriguez et al., 2014)
LEM4	Promote the transcription of cyclin D1	High	(Gao et al., 2018)
SPY1	SPY1 binds to CDK, mediates the phosphorylation of ERK	High	(Ferraiuolo et al., 2017)
SOX9	c-MYC/HDAC5/SOX9 axis	High	(Xue et al., 2019)
SALL2	Activate AKT/mTOR signaling	Low	(Ye et al., 2019)
HIF-1α	Downregulate the expression of ERα	High	(Jogi et al., 2019)

### The Discovery of LEM4 and the Association Between Cell Cycle Regulators and Resistance to Tamoxifen

As a competitive antagonist of estradiol, tamoxifen can bind to estrogen receptors in competition with estradiol and form a stable complex, which inhibits the transcription activity of the estrogen receptor and blocks breast cancer cells in G1 phase to inhibit tumor proliferation. However, tamoxifen has little effect on the cell cycle when cells are treated with tamoxifen alone (Cheng et al., 2017). Previous studies have shown that cyclin D1 and cyclin E are essential for the emergence of tamoxifen resistance in breast cancer cells. Cyclin D1 promotes the progression of the G1–S phase, and tamoxifen can reduce the expression of cyclin D1, which is highly expressed in drug-resistant cells (Viedma-Rodriguez et al., 2014). Based on these mechanisms, scientists have previously proposed many methods to overcome drug resistance, such as the cyclin-dependent kinase (CDK) 4/6 inhibitors palbociclib and ribociclib (Finn et al., 2015; Cristofanilli et al., 2016; Hortobagyi et al., 2016).

The latest research in the last 2 years found that LEM4 (LEM structural protein), which is highly expressed in breast cancer-resistant cells, promotes the transcription of cyclin D1 through ligand-independent activation of receptors. Furthermore, LEM4 interacts with CDK 4/6 and Rb to accelerate the G1–S transition (Gao et al., 2018). Therefore, LEM4 reduces the inhibitory effect of tamoxifen on the G1–S phase transition of breast cancer cells. On the other hand, the existence of LEM4 allows the estrogen receptor to undergo ligand-independent activation in the presence of tamoxifen. LEM4 is expected to be a biological index to predict tamoxifen resistance in ER-positive breast cancer, and targeting LEM4 may be a feasible research direction to overcome tamoxifen resistance in the future.

In addition, Yu et al. (2019) found that cell division cycle associated 8 (CDCA8) may be related to tamoxifen resistance. It is highly expressed in drug-resistant cells. After the CACA8 gene was knocked out, the number of drug-resistant cells in the G1 phase increased, and the drug resistance of the cells to tamoxifen decreased (Yu et al., 2019).

Ferraiuolo et al. (2017) discovered another cell cycle protein, Spy1, which mediates the phosphorylation of ERK under the condition of binding to CDK; the increase in its level is related to tamoxifen resistance.

With the progress of mechanistic research, many new treatments have emerged in recent years. Aspirin (ASA) is a kind of nonsteroidal anti-inflammatory drug that has been used in the treatment of many tumors, including rectal cancer, lung cancer, pancreatic cancer, and breast cancer (Jiang et al., 2020; Wang and Huang, 2020; Wu et al., 2020; Zhang et al., 2020), but whether it is beneficial to the survival of patients is still uncertain. However, the use of aspirin seems to be helpful in overcoming tamoxifen resistance. The expression of cyclin D1 was downregulated, and the number of cells arrested in the G0/G1 phase was increased when tamoxifen was used in combination with ASA. The combination of ASA and tamoxifen can overcome the drug resistance of ER-positive breast cancer cells to tamoxifen (Cheng et al., 2017).

Maqbool et al. (2020) synthesized a novel thiosemicarbazone, DpC. They found that the combination of DpC and tamoxifen effectively reduced cyclin D1, upregulated p27, and inhibited the proliferation of breast cancer cells, which may be helpful to overcome the drug resistance of tamoxifen.

### The Latest Progress in the Relationship Between Autophagy and Resistance to Tamoxifen

Autophagy is the process by which cells engulf their excess proteins or organelles, transport them to lysosomes, and degrade their contents. Their main role is to deal with the stress-induced injury of cells (Antonioli et al., 2017). However, autophagy seems to have two opposing roles in tumor cells. On the one hand, tumor cells can undergo autophagic cell death through self-phagocytosis, after which the cytoskeleton is mostly preserved. On the other hand, autophagy can delay the apoptosis of stressed and damaged cells, and protect their survival (Cook et al., 2011; Sun et al., 2015). Previous studies have shown that autophagy may have a strong relationship with tamoxifen resistance, and it may be an important mechanism of tamoxifen resistance (Gonzalez-Malerva et al., 2011; Nagelkerke et al., 2014), but the relationship between autophagy and tamoxifen resistance is still in the exploratory stage, and the specific mechanism is still unclear.

Recent studies have suggested that autophagy plays a very important role in cell protection. Lysosome-associated membrane protein (LAMP) is an important mediator of the process of autophagy and lysosome fusion. Autophagy was inhibited, and the cells were re-sensitized to tamoxifen after LAMP3 knockdown (Nagelkerke et al., 2014). TAM-R cells have a higher level of autophagy than tamoxifen-sensitive cells, and inhibition of autophagy will improve the efficacy of TAM (Liu et al., 2019). Wang et al. (2019) found that the expression of the H19 gene was enhanced in TAM-R cells and that H19 was significantly related to the enhancement of autophagy in breast cancer cells. Knockout of the H19 gene could make breast cancer cells re-sensitized to tamoxifen.

Why does tamoxifen enhance autophagy and lead to drug resistance? It is well known that tumor cells need a lot of energy to maintain their growth and proliferation, and a significant amount of this energy comes from enhanced glycolysis (Kim and Dang, 2006). The use of tamoxifen has been found to be related to the energy metabolism of cells. It was found that the ATP level of breast cancer cells decreased after tamoxifen treatment. Moreover, the use of tamoxifen could lead to the upregulation of the expression of MTA1, which further destroys mitochondrial function, while drug-resistant cells meet their energy needs through enhanced autophagy (Lee et al., 2018; Das et al., 2019). We speculate that the enhancement of autophagy may be the result of the increased energy demand of tumor cells and the anti-stress response of tumor cells.

Many autophagy-related genes have been discovered, and many autophagy inhibitors have been developed to inhibit tamoxifen resistance. Cheng et al. (2019) found that the use of icariin significantly increased the apoptosis of TAM-R cells; more TAM-R cells remained in the G0/G1 phase, while S phase/G2 phase cells were significantly reduced. At the same time, the expression of cyclin D1, Bcl-2, LC3-1, LC3-II, AGT5, and Beclin-1 were all downregulated. Interestingly, the expression of Beclin-1 downregulates the estrogen signal, which is beneficial to overcoming the resistance to tamoxifen (John et al., 2008). Similarly, Qi et al. (2017) found that autophagy is beneficial to the survival of breast cancer cells, while Z-ligustilide, which inhibits autophagy, may be helpful to overcome the resistance to tamoxifen in breast cancer.

SEL is an antagonist of XOP1. Combined treatment with SEL and 4-OH tamoxifen downregulated the expression of AKT and activated autophagy by blocking the glycolysis pathway, leading to cell death (Kulkoyluoglu-Cotul et al., 2019). Moreover, the degree of autophagy and the expression of autophagy-related genes can be used to judge drug resistance and select the treatment method, which may be helpful for the treatment of ER-positive breast cancer patients in the future.

The relationship between tamoxifen and energy metabolism may become a key research direction in the future, and it is of great significance to control the apoptosis and proliferation of tumor cells and to restore the sensitivity to tamoxifen.

### Progress and Future Direction of Tamoxifen Resistance in Breast Cancer

Endocrine therapy is extremely important for ER-positive breast cancer patients. It mainly includes selective estrogen receptor modulators (SERMs), estrogen receptor downregulated modulators (SERDs), and aromatase inhibitors (AIS). Tamoxifen is one of the SERMs (Ali et al., 2016).

To overcome the resistance to tamoxifen, an increasing number of methods have been studied. ASA can not only reduce drug resistance by blocking G0/G1 phase-resistant cells but also by inhibiting the phosphorylation of AKT (Cheng et al., 2017). Phosphodiesterase 4D (PDE4D) can block cAMP and downstream signaling channels, making cells resistant to tamoxifen. However, the level of cAMP in cells is increased and the phosphorylation level of AKT is decreased after the use of aspirin (Mishra et al., 2018).

In addition, NF-κB has been proven to be related to the resistance of tamoxifen. Li et al. (2019) found that aspirin inhibited the activation of NF-κB signaling, which contributed to overcoming the resistance of cells to targeted therapeutic drugs. Aspirin seems to be a feasible strategy to overcome tamoxifen resistance, and it is expected to provide a new direction to breast cancer treatment. In addition to ASA combined with tamoxifen, proteasome inhibitors (PIs) combined with endocrine therapy have also been proven to be beneficial to the sensitization of tamoxifen-resistant cells (Maynadier et al., 2016; Cheng et al., 2017).

Inhibiting kinases in the RTK pathway to overcome drug resistance is also considered to be a viable approach. For example, gefitinib, perifosine, or GnRH-I and GnRH-II analogs were used to inhibit AKT expression (Block et al., 2012). Giordano et al. (2011) found that the primary bile acid chenodeoxycholic acid (CDCA) can activate the farnesoid X receptor (FXR) and inhibit the expression of HER2. Quercetin has also been found to restore the sensitivity to tamoxifen by mediating the upregulation of ERα and the downregulation of HER-2 (Wang et al., 2015).

The combination of tamoxifen and gefitinib promoted the apoptosis of drug-resistant cells. Gefitinib inhibited the downregulation of ERα by EGFR and restored the sensitivity of cells to tamoxifen to a certain extent (Jeong et al., 2019). Interestingly, another study showed that gefitinib has no effect on the activity of breast cancer-resistant cells, while neratinib, another EGFR inhibitor, induced the apoptosis of resistant cells by inhibiting the EGFR and HER2 signaling pathways (Kim et al., 2015). In addition, the use of dichloroacetate can overcome tamoxifen resistance by downregulating EGFR (Woo et al., 2016). Therefore, further study is needed on the effect of EGFR inhibitors on tamoxifen-resistant cells.

Peptidyl-prolyl isomerase Pin1 participates in the development of drug resistance by inducing E2F-4. Interestingly, all-trans retinoic acid (ATRA), an inhibitor of Pin1, inhibits the drug resistance of cells mainly by inhibiting the ERK 1/2 and AKT pathways (Huang et al., 2019).

Inhibition of epithelial–mesenchymal transition (EMT)-like phenomena is also a direction to take to overcome drug resistance. In addition to LDHA inhibiting EMT-like phenomena, resveratrol can also inhibit EMT by inhibiting TGF-β and overcoming tamoxifen resistance. Interestingly, EGFR activation is also related to EMT-like phenotype change, which confirms that inhibition of EMT contributes to overcome tamoxifen resistance (Zuo et al., 2011; Shi et al., 2013; Das et al., 2019). The drugs that overcome tamoxifen resistance mentioned in this review are summarized in Table 2.

**TABLE 2:** Summary of recent studies of drugs that may be helpful in improving tamoxifen resistance.

Medicine	Therapeutic mechanism	Ref
ASA	block cell cycle in G0/G1 phase	(Cheng et al., 2017)
	target PDE4D/cAMP/ER stress axis	(Mishra et al., 2018)
	suppressed NF-κB signaling pathway	(Li et al., 2019)
Gefitinib/Perifosine/ analogs of GnRH-Ⅰ/Ⅱ	inhibit erbB and AKT signaling	(Block et al., 2012)
CDCA	inhibit HER2 expression	(Giordano et al., 2011)
Quercetin	mediate upregulation of ERα and downregulation of HER2	(Wang et al., 2015)
Neratinib	inhibit EGFR and HER2 signaling pathway	(Kim et al., 2015)
Dichloroacetate	downregulate EGFR expression	(Woo et al., 2016)
ATRA	inhibit the activation of ERK 1/2 and AKT	(Huang et al., 2019)
Resveratrol	reduce endogenous TGF-β production and reverse EMT	(Shi et al., 2013)
DpC	inhibit the expression of cyclin D1 and ERα	(Maqbool et al., 2020)

With the advances in science and technology, some new approaches have been developed to improve tamoxifen sensitivity. For example, the application of nanotechnology (Guney Eskiler et al., 2018; Kumar et al., 2018) and the benefits of cold atmospheric plasma (CAP) in overcoming drug resistance, etc. (Lee et al., 2017).

Early judgments about the possible efficacy of endocrine therapy is of great significance in clinical treatment. Therefore, some prognostic markers suggesting tamoxifen resistance have been identified (Putluri et al., 2014; Elias et al., 2015; Gwak et al., 2017; Shimoda et al., 2017; Gong et al., 2018). The discovery of these markers is conducive to making early judgments about endocrine therapy efficacy and predictions of recurrence, which is helpful for doctors when making appropriate changes to the treatment strategy.

## Conclusions and Prospects

Tamoxifen plays an important role in ER-positive breast cancer patients. However, drug resistance limits its efficacy, illustrating the importance of overcoming tamoxifen resistance in breast cancer. Most methods to overcome breast cancer resistance are based on the mechanism of drug resistance, such as inhibition of the RTK pathway, upregulation of ERα36, and blocking protective autophagy, cell cycle regulators and EMT-like phenomenon. In addition, some new methods have broadened the field of vision to overcome the drug resistance of tamoxifen. For example, some drugs combined with tamoxifen can inhibit the development of drug resistance, and the development of some new technologies is conducive to reducing the drug resistance of tamoxifen, and some prognostic markers of tamoxifen resistance have been discovered.

Research on the relationship between autophagy, cell cycle regulators, and resistance to tamoxifen has made great progress in recent years. The enhanced autophagy in drug-resistant cells is mainly due to the destruction of mitochondrial function caused by tamoxifen, and drug-resistant cells meet their energy demand through autophagy. The methods to overcome drug resistance according to the autophagy mechanism are mainly limited in the current research to the inhibition of autophagy by autophagy inhibitors.

In addition to continuing to look for better autophagy inhibitors to overcome the resistance, we hypothesized that tamoxifen combined with other drugs that protect mitochondrial function can prevent enhanced autophagy and overcome the drug resistance of tamoxifen. This is a new idea to improve the drug resistance of tamoxifen, and there is little research in this area.

Moreover, by detecting the level of autophagy and the expression of autophagy-related genes, the level of cell resistance can be judged, and treatment can be formulated and changed accordingly, which may improve the clinical treatment of breast cancer.

Targeting LEM4 is a feasible research direction to overcome tamoxifen resistance in the future. It has been proven that the high expression of LEM4 in drug-resistant cells is an important mechanism involved in the attenuation of the inhibitory effect of tamoxifen on the G1–S transition. Targeting LEM4 will play a significant role in overcoming tamoxifen resistance.

Overall, the main direction to overcome tamoxifen resistance in the future is not limited to inhibiting the expression of pathways related to tamoxifen resistance but may focus more on cyclins related to tamoxifen resistance, targeting LEM4 and inhibiting autophagy.

## Author Contributions

Conception: JY, JZ, and JH. Collection and assembly of data: All authors; Data analysis and interpretation: JY, JZ, KD, and RZ; Manuscript writing: JY and JZ; Final approval of manuscript: All authors.

## Conflict of Interest

The authors declare that the research was conducted in the absence of any commercial or financial relationships that could be construed as a potential conflict of interest.
